# A Systematic Search and Review of Questionnaires Measuring Individual psychosocial Factors Predicting Return to Work After Musculoskeletal and Common Mental Disorders

**DOI:** 10.1007/s10926-020-09935-6

**Published:** 2020-12-23

**Authors:** Andrea Gragnano, Patrizia Villotti, Christian Larivière, Alessia Negrini, Marc Corbière

**Affiliations:** 1 Department of Psychology, Università degli Studi di Milano-Bicocca, Piazza dell’Ateneo Nuovo, 1, Milan, 20126 Italy; 2 Career counselling - Department of Education and Pedagogy, Université du Québec à Montréal, Montreal, Canada; 3 Institut de Recherche Robert-Sauvé en Santé et en Sécurité du Travail, Montreal, Canada; 4 Centre de recherche de l’Institut Universitaire en Santé Mentale de Montréal, Montreal, Canada

**Keywords:** Return to work, Psychosocial factors, Measurement tools, Common mental disorders, Musculoskeletal disorders

## Abstract

*Purpose* Individual psychosocial factors are crucial in the return to work (RTW) process of workers with musculoskeletal disorders (MSDs) and common mental disorders (CMDs). However, the quality and validity of the questionnaires used to measure these factors have rarely been investigated. The present systematic search and literature review aims at identifying, categorizing, and evaluating the questionnaires (measurement tools) used to measure individual psychosocial factors related to the perception of the personal condition and motivation to RTW that are predictive of successful RTW among workers with MSDs or CMDs. *Methods* Through a systematic search on PubMed, Web of Science, and PsycINFO library databases and grey literature, we identified the individual psychosocial factors predictive of successful RTW among these workers. Then, we retrieved the questionnaires used to measure these factors. Finally, we searched for articles validating these questionnaires to describe them exhaustively from a psychometric and practical point of view. *Results:* The review included 76 studies from an initial pool of 2263 articles. Three common significant predictors of RTW after MSDs and CMDs emerged (i.e., RTW expectations, RTW self-efficacy, and work ability), two significant predictors of RTW after MSDs only (i.e., work involvement and the self-perceived connection between health and job), and two significant predictors of RTW after CMDs only (i.e., optimism and pessimism). We analyzed 30 questionnaires, including eight multiple-item scales and 22 single-item measures. Based on their psychometric and practical properties, we evaluated one of the eight multiple-item scales as questionable and five as excellent. *Conclusions:* With some exceptions (i.e., self-efficacy), the tools used to measure individual psychosocial factors show moderate to considerable room for improvement.

**Electronic supplementary material:**

The online version of this article (10.1007/s10926-020-09935-6) contains supplementary material, which is available to authorized users.

## Introduction

Musculoskeletal disorders (MSDs, such as low back pain) and common mental disorders (CMDs, such as depression) represent prominent causes of sickness absence and work disability worldwide [1]. An average of 6% of the working-age population in OECD countries have disability benefits as their primary income, and in some countries, this percentage doubles [2]. MSDs and CMDs account for 42% of the Years Lived with Disability (YLD) globally, with 21% each [3]. The employment rate of people with disabilities in OECD countries is on average 40% lower than that for the general population, and the unemployment rate is twice the overall level [2]. Work is crucial for people with disabilities, also because it promotes mental and physical recovery, functional abilities, and social participation [4]. In summary, MSDs and CMDs place hefty economic, social, and personal burdens on society.

It is therefore necessary to promote the return to work (RTW) of people with these disorders. To this end, there is well-established literature investigating the factors that facilitate and hinder the RTW. The RTW is regarded as a multidimensional process influenced mainly by psychosocial determinants (e.g., RTW expectations and job strain) and macrosystem variables (e.g., the healthcare and workplace systems) and, to a much lesser extent, by traditional medical indicators (e.g., symptom severity and prognosis) [5]. Reviews have highlighted that two categories of psychosocial factors are particularly relevant yet understudied for the RTW. The first category includes the organizational psychosocial factors associated with the genesis of strain, whereas the second includes individual psychosocial factors related to the perception of the personal condition and motivation to RTW [6–8]. A serious limitation of the study of psychosocial factors is the considerable heterogeneity in the definition and measurement of the psychosocial predictors among different studies [7, 9]. This limitation is associated with a lack of sufficiently validated measurement tools [7, 9]. Ultimately, this situation means that researchers and clinicians face a fragmented and inconsistent scientific literature when planning to measure individual and organizational psychosocial factors for a new study, clinical purposes, or international comparisons. It is therefore urgent to start identifying which psychosocial factors are relevant to the RTW process and how they are measured in order to identify both the pros and cons of existing measurement tools and gaps in the literature.

The present systematic search and literature review aims at identifying, categorizing, and evaluating the questionnaires used to measure the individual psychosocial factors related to the perception of the personal condition and motivation to RTW (e.g., RTW self-efficacy and RTW motivation, hereinafter referred to as “individual psychosocial factors”) that are predictive of RTW among workers with MSDs or CMDs. To this end, it is necessary first to identify the individual psychosocial factors predictive of successful RTW and then to review questionnaires in terms of their psychometric properties and practical information useful for clinicians. The same type of review, but concerning *organizational* work-related psychosocial factors predictive of RTW, has been conducted, and the results have been published elsewhere [10].

## Methods

We adopted a two-phase search strategy. The first phase involved identifying the individual psychosocial factors predictive of successful RTW among workers with MSDs or CMDs and the related questionnaires used. In this review we considered two primary indicators of success in returning to work: (a) the probability of being back at work at the time of study follow-up, or (b) the time to return to the workplace, meaning the duration of work absence since the first day of absence due to the MSD or CMD. The review included both studies examining RTW as a single event and studies examining sustainable RTW (i.e., RTW for a minimum number of days). The second phase involved a search for articles that validated the questionnaire in order to describe them exhaustively from a psychometric and practical point of view.

### Identification of the Individual Psychosocial Factors

#### Search Strategy

A systematic literature search was conducted in PubMed, PsycInfo, and Web of Science databases from January 1998 to January 2018 (20 years). We also performed a complementary search of non-indexed literature (Google Scholar) and identified additional articles from the bibliographic references in relevant articles. Four groups of keywords, combined by the Boolean operator *and*, were used. These groups were (i) outcome of interest (e.g., return to work *or* work participation *or* work reintegration), (ii) the work status (e.g., sickness *or* absence *or* off-work *or* disability *or* rehabilitation), (iii) psychosocial factors (e.g., work-ability *or* self-efficacy *or* expectation *or* motivation), and  (iv) study type (e.g., longitudinal *or* prospective *or* wave study). A further group was added, combined with the Boolean operator *and not*, to exclude samples not of interest (e.g., stroke *or* brain injury *or* sclerosis *or* child).

Articles were selected if they met the following inclusion criteria: (1) they were prospective cohort studies; (2) study subjects had an MSD or a CMD or, for mixed population studies, at least two thirds (≥ 67%) of the study sample consisted of people suffering from an MSD and/or a CMD; (3) study subjects were workers on sick leave at the time of data collection (i.e., baseline), or if that was not the case, the condition of those not on sick leave or not employed was controlled for in the analyses; (4) the studies analyzed one of the two indicators of success in returning to work previously defined; (5) one or more individual psychosocial factors measured and tested as predictors of the outcome in multivariate regressions controlling for at least age and sex/gender; and (6) studies were written in English or French. The exclusion criteria were as follows: (1) articles were literature reviews, case studies, qualitative studies, or cross-sectional studies; and (2) study subjects were sick-listed workers with unspecified work disability.

Articles were selected first based on the title and abstract, then based on the full text. The article selection based on title and abstract was performed by three trained reviewers, PhD or Master’s students. Two additional independent reviewers (the first two authors) double-checked approximately 30% of the references. In case of discrepancy, an agreement was reached through discussion based on the information available in the title and abstract. The selection based on the full text was performed by one researcher (the first author). If the inclusion of an article was uncertain, another researcher (the second author) read the full text to reach a joint decision. When disagreement occurred after these two readings, a third researcher (last author) was consulted to reach full agreement.

#### Data Extraction

For each study selected, we gathered information about the individual psychosocial factors considered. We listed the population in which they were tested (i.e., MSD, CMD, or mixed), the crude and adjusted effects, and the type of outcomes. From this information, adopting the “best-evidence synthesis procedure” [11], we classified the individual factors as having a “limited”, “moderate”, “strong”, “insufficient”, or “inconsistent” level of evidence of their ability to predict RTW in the two populations considered separately. The level of evidence was attributed counting the number of multivariate effects tested that were statistically significant (*p* < 0.05) with a positive relationship with the outcome, statistically significant with a negative relationship with the outcome, and not statistically significant. At first, factors were scrutinized for consistency of the effects, that is, if the significant effects were in the same positive or negative direction. A factor was labeled as inconsistent if the ratio of significant positive effects to total (positive and negative) significant effects was between 0.45 and 0.65. If the factor was consistent, we determined the level of evidence supporting its predictivity based on *X*, where *X* equalled the ratio of significant positive (or negative) effects to total significant and non-significant effects. The rules were adapted from Gragnano et al. [7].

The level of evidence was classified as (o) insufficient, when *X* < 0.60; (i) limited, when only one significant effect (positive or negative) was found, or 0.60 ≤ *X* < 0.65; (ii) moderate, when only two significant effects in the same direction were found, or 0.65 ≤ *X* < 0.80; or (iii) strong, when 0.80 ≤ *X* ≤ 1.00.

We evaluated the number of effects separately for MSDs and CMDs. The effect tested in a sample consisting of both MSDs and CMDs was counted both in the evaluation of MSDs and that of CMDs. To be considered in the second step of identification of the measurement tools, a factor had to have a level of evidence classified at least as “moderate” for MSDs or CMDs.

It should be noted that the classifiers “insufficient”, “limited,” “moderate”, and “strong” did not pertain to the effect size of each factor. These classifiers represented the quantity (number of studies) and consistency (negative or positive relationship) of the effects (statistically significant and not) of each factor on the RTW success.

### Identification and Description of the Measurement Tools

#### Search Strategy

For each factor predictive of RTW with at least a moderate level of evidence, we considered the studies that reported a multivariate statistically significant effect of that factor. For all these studies, we listed the tools used to measure the factor. For all the extracted questionnaires, we searched in the references and PubMed, PsycInfo, and Web of Science databases for articles validating the tools. From all these articles, we collected psychometric properties and practical information useful for clinicians.

We considered the following psychometric characteristics: (i) predictive validity; (ii) face validity; (iii) construct validity; (iv) internal consistency; (v) convergent validity; and (vi) test–retest reliability. All the measurement tools had predictive validity, as it was a requirement for inclusion in the list of tools. We reported information about the crude and adjusted effects detected with that tool. Face validity was estimated through qualitative inspection of the items used to measure a specific factor/concept in the measurement tool. Construct validity was evaluated positively if a factor analysis of the structure of the measure was found to exist. Internal consistency was evaluated positively if Cronbach’s alphas ranged between 0.70 and 0.95. Convergent validity was evaluated by significant and positive correlations with theoretically similar concepts. Test–retest reliability was rated positively when repeated testing of the same condition yielded comparable results (correlation coefficients higher than 0.60) [12].

The practical characteristics considered were (i) time required to complete the questionnaire, (ii) difficulties for the clinician in calculating the final score, (iii) fee or training needed for administering the questionnaire and interpreting the scores, and (iv) availability of the measurement tool. More specifically, the time required to complete the questionnaire was favorably rated if questionnaires had fewer than eight items. The final score was considered easy to calculate if it only required summing the items’ scores. The final score was considered difficult to obtain when a more complex formula was needed or when reversed items were present. The absence of a fee to pay and of training to follow on use of the measurement tool was evaluated positively. Instrument availability was evaluated positively if an English version of the measurement tool was easily available.

Based on how many psychometric and practical criteria the measurement tool met, we adopted rules for the evaluation (Table 1). Psychometric properties were evaluated for multiple-item scales. Therefore, single-item measures did not undergo a summary evaluation.

**Table 1:** Rules for the summary evaluation of measurements tools

	Six psychometric criteria			
	N of criteria positively met	5–6	3–4	≤ 2
Four practical criteria	4	Excellent	Excellent	Questionable
	3	Excellent	Good	Questionable
	≤ 2	Excellent	Good	Questionable

## Results

**Fig. 1:**
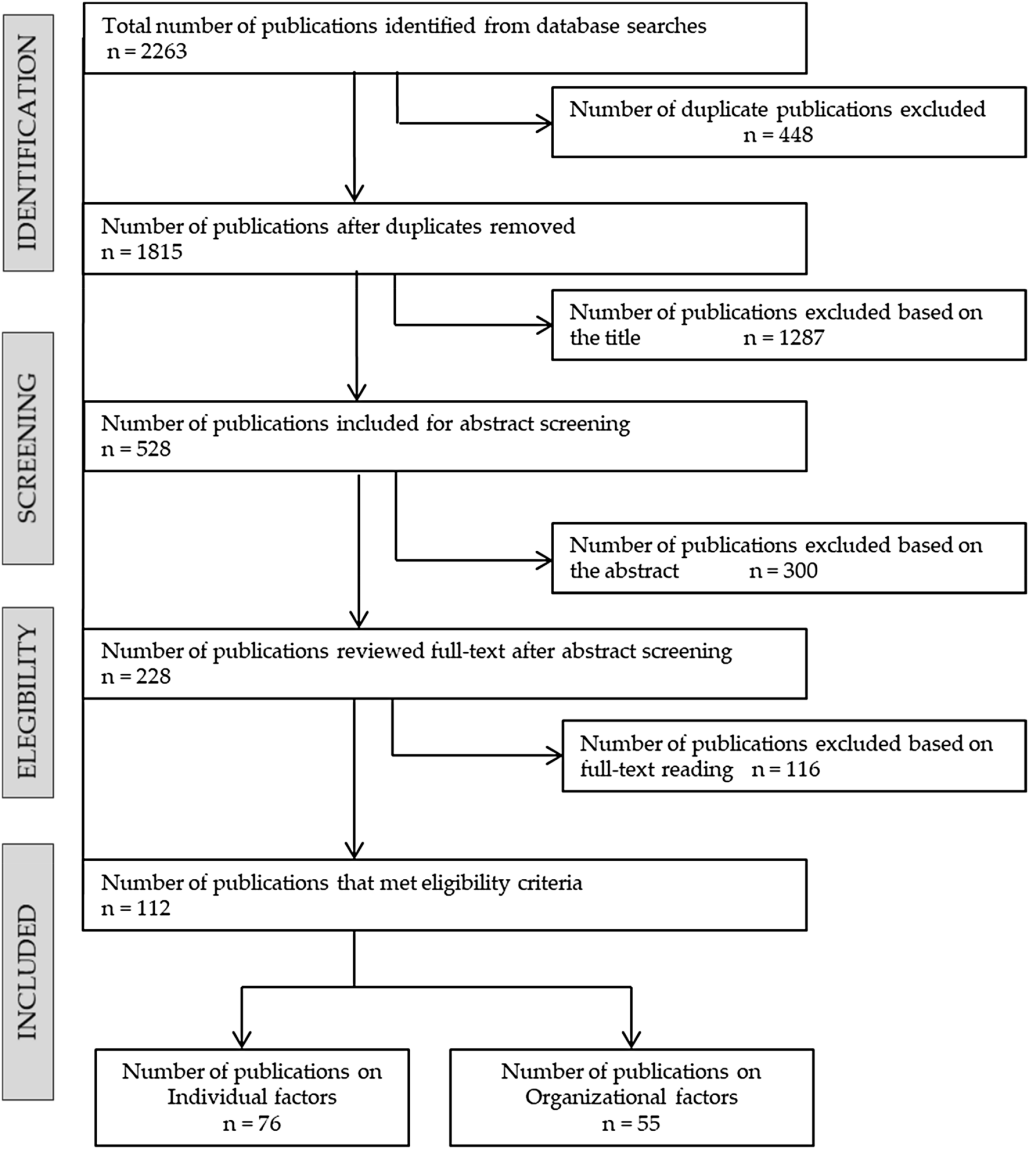
Results of the search strategy. The same publication can investigate both individual and organizational factors; therefore, the sum of the publications on individual and organizational factors is higher than the number of publications that met eligibility criteria

Figure 1 shows the results of the search strategy. In this study, we considered the individual psychosocial factors predictive of RTW. Villotti et al. published a similar review for organizational psychosocial factors [10]. The selection procedure in our study yielded 76 studies investigating individual psychosocial factors among people with an MSD and/or a CMD.

Among the individual psychosocial variables examined in these 76 studies, three were common significant predictors of RTW after MSDs and CMDs, two were significant predictors of RTW after MSDs only, and two others were significant predictors of RTW after CMDs only. Table 2 reports these predictors along with the references. We found a total of 35 effects for MSDs and 19 for CMDs. With regards to MSDs, RTW expectations, RTW self-efficacy, and work ability emerged as strong predictors of RTW, whereas work involvement and the self-perceived connection between health and job emerged as limited predictors. Apart from the self-perceived connection between health and job, these predictors were facilitators of the RTW process. With regards to CMDs, RTW self-efficacy was the only strong predictor of RTW, RTW expectations was a moderate predictor, and work ability, optimism, and pessimism were limited predictors of RTW. Apart from pessimism, these predictors were facilitators of the RTW process.

**Table 2:** Individual psychosocial predictors of RTW after MSDs and CMDs

Factor	MSD		CMD		Level of evidence^d^	
	N and direction of the effects	References	N and quality of results	References	MSDs	CMDs
Return to work expectations	20 +	[14–18, 20–25, 27–34] ^a^	6 +	[19, 21, 23, 25, 26, 35]	Strong + 0.95	Moderate + 0.75
	1 ns	[14]^a^	2 ns	[32, 42]		
RTW self-efficacy	4 +	[38, 47] [36, 37, 46]^b^	3 +	[40, 41, 47]	Strong + 1.00	Strong + 1.00
Work ability	8 +	[67]^c^, [25, 32, 33, 49, 53, 61, 68]	3 +	[52, 68, 69]	Strong + 0.89	Limited + 0.60
	1 ns	[54]	2 ns	[25, 32]		
Work involvement	1 +	[70]	1 ns	[70]	Limited +	Insufficient
Self-perceived connection between health and job	1 −	[25]	–	–	Limited −	–
Optimism	–	–	1 +	[71]	–	Limited +
Pessimism	–	–	1 −	[71]	–	Limited −

^a^The same study [14] provided two different results because RTW was measured in two different population (i.e., low back pain and other msd)

^b^Same sample in Richard et al. [36] and Dionne et al. [37]

^c^No specific tool mentioned in the article or retrievable from the references

^d^Level of evidence is the ratio of significant positive (or negative) effects to total significant and non-significant effects

### RTW Expectations

RTW expectations refers to the worker’s expectations of how difficult/likely/long the process of employment resumption will be. RTW expectations are a strong predictor of RTW after MSDs and a moderate one after CMDs (Table 2). The less difficult, more likely, or shorter the RTW process is expected to be, the more frequently this positive expectation will materialize. Table 3 describes the tools with predictive validity used to measure RTW expectations, along with their summary evaluation. Only one scale composed of more than one item was used. This scale was the Work-Related Recovery Expectations Questionnaire, whichwas used in three studies [13–15]. Following the rules for the summary evaluation of measurement tools, this questionnaire was evaluated as questionable because it met two out of six and three out of four psychometric and practical criteria, respectively. Nineteen single-item measures assessed RTW expectations [16–35]. No summary evaluation was performed of these tools because the psychometric criteria were not applicable. These single-item measures of RTW expectations can be classified as single-item measures with and without a time reference. The first group was subdivided into single-item measures with a time frame in terms of months (six measurement tools [16, 17, 28–32]) or in terms of weeks (three measurement tools [33–35]). The second group was subdivided into single-item measures that ask the respondents to estimate their confidence in the RTW (one measurement tool [18]), work ability (four measurement tools [19–22]), or the time they will take to RTW (five measurement tools [23–27]). Tables 4 and 5 (Online resources 1 and 2) report the psychometric and practical characteristics of the tools used to measure RTW expectations. Table 6 (Online resource 3) reports detailed information about the tools’ predictive validity.

**Table 3:** Tools description and evaluation

Tool	References	Country	Total N	Population	Structure	Psychometric quality	Practical quality	Classification
RTW expectation								
Work-related recovery expectations questionnaire	[14, 15, 72]	Alberta, Canada	1210	MSD	3 statements. Agreement expressed on a five-point Likert scale. First item response direction is reversed as compared with the other two. Lower score represents better expectations	2/6	3/4	Questionable
Single item								
Question with no time reference (confidence in RTW)								
To what extent do you think you will return to work?	[18]	Norway	567	LBP	4 response options: 1 (to a low degree); 2 (to a certain degree); 3 (to a high degree); 4 (do not know)	–	4/4	Not applicable
Question with no time reference (work ability)								
Do you expect to be able to return to your workplace?	[19]	Denmark	644	CMD	2 answer options: *Yes*–*No*	–	4/4	Not applicable
When do you think you will be able to work fulltime again?	[20]	Nederland	299	LBP	8 response options: 1 (within 1 week); 2 (within 1 month); 3 (within 1–2 months); 4 (within 2–3 months); 5 (within 3–6 months); 6 (within 6–12 months); 7 (beyond 12 months); 8 (no idea)	–	4/4	Not applicable
What is your opinion about your work ability in the long-term?	[21]	Sweden	525	MSD, CMD	5 response options: 1 (I will be working in my profession with the same working hours as before); 2 (I will be working in my profession but with reduced working hours); 3 (I will be working in another profession but with the same working hours as before); 4 (I will be working in another profession but with reduced working hours); 5 (I will not be able to work anymore). Answers between 1 and 4 codified as 1 (Positive prediction); answers 5 codified as 0 (Negative prediction)	–	4/4	Not applicable
What do you believe, honestly, is the probability that you will become so much better that you will be able to work at some time in the future?	[22]	Sweden	122	MSD	(Very improbable—Improbable—Rather improbable = Low self-prediction)—(Rather probable—probable—Very probable = High self-prediction)	–	4/4	Not applicable
Question with no time reference (expected duration)								
For how long do you believe you will be sick listed from today?	[23]	Norway	217	MSD & CMD	6 response options: not at all, less than 1 month, 1–2 months, 2–4 months, 4–10 months, more than 10 months	–	4/4	Not applicable
When do you think you will be able to work full-time again?	[24]	Netherlands	276	MSD, self-employed	The answering categories were combined into within one month; more than one month/never; and no idea	–	4/4	Not applicable
Approximately how long do you think you will need to return to the job you had before you went on sick leave? (we understand that this question is difficult to answer, please try to give an answer, even if it is only approximate)	[25]	Spain	663	MSD, CMD	7 response options: Less than 1 week—Between 1 and 4 weeks—Between 1 and 3 months—Between 4 and 6 months—Over 6 months—I will never be able to perform the job I used to before—I do not know, I have no idea how long I will take to recover. Collapsed into: < 1 mo.; 1–3 mos.; > 3 mos.; and I will never be and I don’t know	–	4/4	Not applicable
How many months do you think it will take you to fully return to work?	[26]	Nederland	168	CMD	Response format not specified. Answers were dichotomized into expected duration ≤ 3 months or > 3 months	–	4/4	Not applicable
Expected duration of sick leave	[27]	Netherlands	615	LBP	2 response options: From one to 10 days—10 days or more	–	4/4	Not applicable
Question with a time reference (months)								
How likely is it that within the next month you will have resumed some form of employment?	[16]	Quebec, Canada	109	MSD	Participants indicated their responses on a percentage scale from not at all likely (0%) to extremely likely (100%). Cut off 62.5% determined with a ROC analysis	–	4/4	Not applicable
Do you think you will be back to your normal work within 3 months?	[17]	Quebec, Canada	1007	BP	3 response options: Yes—No—Undecided, don’t know	–	4/4	Not applicable
Participants rated their certainty they would be working in 6 months	[28, 29]	US	1068 + 899	LBP, CTS	11 response options from 0 (not at all certain) to 10 (extremely certain) recoded as: 0 (Very low); 1–4 (Low); 5–7 (Moderate); 8–9 (High); 10 (Very high)	–	3/4	Not applicable
Do you expect to return work within 6 months?	[30]	Belgium	346	LBP	11 response options from 0 (No chance) to 10 (Very large chance)	–	4/4	Not applicable
In your estimation, what are the chances that you will be able to resume work in 6 months?	[31]	Belgium	186	LBP	11 response options from 0 (No chance) to 10 (Very large chance). Recoded as 10 (Very sure to RTW within 6 months); < 10 (not very sure to RTW within 6 months)	–	4/4	Not applicable
In your estimation, what are the chances that you will be working in 6 months?	[32]	Sweden	699	MSD, CMD	5 response options from 1 (Very good chance) to 5 (very little chance)	–	4/4	Not applicable
Question with a time reference (weeks)								
What do you believe your situation concerning certified sickness absence will be in 4 weeks?	[33]	Norway	190	LBP	3 response options: Probably full—time certification—Probably part-time certification—Probably returned to work. Dichotomized into Returned to work and Continued certification	–	4/4	Not applicable
Expectations regarding RTW were measured by asking whether they expected to return to work within the next few weeks or not	[34]	Norway	246	LBP	Wording of question not available Response option: Yes/No	–	3/4	Not applicable
I expect to be back at work within the next few weeks	[35]	Norway	241	CMD	5 response options from *Strongly agree* to *Strongly disagree*	–	4/4	Not applicable
RTW self-efficacy								
Self-efficacy for return to work questionnaire	[36,37]^a^ and Appendix of Dionne et al. [17]^a^	Quebec, Canada	1007	MSD	Scale developed according to Bandura’s guidelines. It measures the subject’s perception of his or her capacity to do his or her regular work despite some specific obstacles (i.e.: pain, lack of support, work pressure, difficulties in commuting). Obstacles were identified in a previous qualitative study	4/6	2/4	Good
Return-to-Work Self-Efficacy Scale	[38, 39]	Ontario, Canada	551 + 419	MSD	Three subdomains: (1) the RTWSE Pain subscale = 4 items, i.e. the ability to cope with pain (pain-tolerate, pain-prevent, pain manage), (2) the RTWSE Supervisor subscale = 4 items, i.e. the ability to obtain help from supervisor and (3) the RTWSE Co-workers subscale = 2 items, i.e. the ability to obtain help from coworkers	5/6	3/4	Excellent
Return-to-Work Self-Efficacy Scale-19	[46]	Texas, USA	399	LBP	Three factors: (1) meeting job demands = 7 items, (2) modifying job tasks = 8 items, and (3) communicating needs to others = 4 items. Respondents’ level of confidence about overcoming several return-to-work barriers was reported on a scale ranging from 0 to 10 Three groups formed according to total RTWSE: low self-efficacy (< 5), medium self-efficacy (scores from 5–7.5), and high self-efficacy (> 7.5)	6/6	2/4	Excellent
Return-to-work self-efficacy questionnaire	[40–45]^b^	Netherlands	1855 (+ 2744 all cause sickness absence)	CMD	One factor. 11 items covering several problem CMD workers can face when returning to work. Participants were asked to respond to statements about their jobs, imagining that they would start working their full contract hours again tomorrow (in their present emotional state/ state of mind)	6/6	2/4	Excellent
Return-to-Work Obstacles and Self-Efficacy Scale	[47]	Quebec, Canada	206 + 157	MSD and CMD	Presented in two parts that appear on the same page: perceived obstacles to RTW (Part A) and self-efficacy beliefs about overcoming them (Part B) in 10 different dimensions (factors)	5/6	2/4	Excellent
Work ability								
Graded reduced work ability scale	[49, 61]	Norway	260 + 457	MSD	The scale consists of 5 items grading the perceived working capacity of patients in relation to the complaints for which they requested sick leave, rated on 5-point scales, and 1 item measuring other symptoms, rated with a 3-point scale (*yes*, *no*, *don't know*). Hagen et al. [49] analyzed separately only 3 of the 6 items (reduced ability to work, the belief work will aggravate condition, and other complaints)	4/6	3/4	Good
Work Ability Index	[50, 68, 69]	Finland (but used worldwide)	273 + 87	MSD and CMD	10 items with different rating scales. The WAI covers the following dimensions of individuals: (1) their current work ability compared with their lifetime best; (2) their work ability in relation to the demands of the job; (3) the number of diagnosed illnesses or limiting conditions from which they suffer; (4) their estimated impairment owing to diseases; (5) the amount of sick leave they have taken during the last year; (6) their own prognosis of their work ability in 2 years; and (7) mental resources	5/6	2/4	Excellent
Single item								
The single-item WAI question Assume that your work ability at its best has a value of 10 points. How many points would you give your current work ability? (0 means that you cannot currently work at all)	[32, 52–54]	Denmark, Nederland, Sweden	223 + 72 + 699 + 328	MSD and CMD	11 response options from 0 (completely unable to work) to 10 (work ability at its lifetime best)	Ahlstrom et al. [55] compared the full version of the scale with the single item. Results suggest the single item may be a good alternative to the full scale	4/4	Not applicable
To what degree does your back disorder reduce your ability to perform your ordinary work today?	[33]	Norway	190	LBP	5 response options: 1 (hardly reduced at all), 2 (not much reduced), 3 (moderately reduced), 4 (much reduced), 5 (very much reduced). Collapsed into: 1–3 = 1 (moderately reduced), 4 = 2 (much reduced), 5 = 3 (very much reduced)	-	4/4	Not applicable
To what extent do you feel that, at this current moment in time, your ability to perform your usual job is lower than before?	[25]	Spain	663	MSD & CMD (tested but n.s.)	11 response options from 0 (hardly reduced at all) to 10 (extremely reduced). Values recoded into three categories: 0–3 (not at all or slightly reduced work ability), 4–6 (moderately reduced), and 7–10 (very or extremely reduced)	-	4/4	Not applicable

*LBP* Low back pain, *CTS* carpal tunnel syndrome

^a^Same sample in Richard et al. [36] and Dionne et al. [17, 37]

Two studies [42, 45] were not included in the count of evidence because the sample was made by all-cause sickness absence or because predictivity was tested only univariately

### RTW Self-efficacy

RTW self-efficacy indicates the belief the workers have in their ability to complete the RTW process successfully and to overcome possible obstacles during the RTW process. With four significant effects for MSDs and three for CMDs, RTW self-efficacy is a strong predictor of RTW after both MSDs and CMDs (Table 2). More specifically, a higher RTW self-efficacy is a facilitator of the RTW process. Table 3 describes the five scales with predictive validity used to measure RTW self-efficacy, along with their summary evaluation. The Self-efficacy for Return to Work Questionnaire [17, 36, 37] was evaluated as good. It did not meet two psychometric (i.e., construct validity and test–retest reliability) and two practical (i.e., length—8 items, and final score computation—not clearly defined) criteria. The other four scales were evaluated as excellent.

The Return-to-Work Self-Efficacy Scale [38, 39] failed to meet only one psychometric criterion (i.e., test–retest reliability) and one practical criterion (i.e., length; it consists of ten items). The Return-to-work Self-Efficacy Questionnaire [40–45] and the Return-to-Work Self-Efficacy Scale-19 [46] met all the psychometric criteria but did not meet two practical criteria (i.e., length and final score computation—some basic calculations needed to be made). Finally, the Return-to-Work Obstacles and Self-Efficacy Scale [ ROSES, 47] did not meet one psychometric (i.e., convergent validity) and two practical criteria (i.e., length—46 items, and final score computation). The length of ROSES is due, not only to the inclusion of two concepts at the same time (i.e., barriers to return to work and self-efficacy to overcome these RTW barriers), but also to the inclusion of 10 conceptual subscales (e.g., job demands). Tables 4 and 5 (Online resources 1 and 2) report the psychometric and practical characteristics of the tools used to measure RTW self-efficacy. Table 6 (Online resource 3) reports detailed information about the tools’ predictive validity.

### Work Ability

In this context, work ability refers to the worker’s evaluation of his/her personal work capability in light of his/her health condition and the work demands. Work ability is a strong predictor of RTW after MSDs and a limited one after CMDs (Table 2). Increased work ability facilitates the return to work. Table 3 describes the measurement tools with predictive validity used to measure work ability, along with their summary evaluation. Two scales—Graded Reduced Work Scale [48, 49] and the Work Ability Index [50]—and three single-item measures were used. The Graded Reduced Work Ability Scale and the Work Ability Index were evaluated as good and excellent, respectively. Of the six psychometric criteria considered, the Graded Reduced Work Ability Scale scored four; there was no evidence of convergent and test–retest validity. The Work Ability Index scored five because a two-factor solution seemed to perform better than the hypothesized one-factor model [51]. Of the four practical criteria considered, the Graded Reduced Work Ability scale scored three; final score computation was not clearly defined, whereas the Work Ability Index scored two because of the length and of a complex final score computation. One single-item measure, the Single-Item WAI question [32, 52–54], consisted of one item from the Work Ability Index [50]. Unlike all the other single-item measures considered in this review, for the Single-Item WAI question, one study was found that compared the performance on the single item with that on the full scale, suggesting that the single item may be a good alternative to the full scale [55]. For this reason, the Single-Item WAI question is also reported in Table 4 (Online resource 1), even if a final score was not computed. The Single-Item WAI question asks the respondent to rate the current work ability compared to the best possible work ability. The other two single-item instruments asked how much the work ability is reduced by the “back disorders” [33] or at this “current moment in time” [25]. Tables 4 and 5 (Online resources 1 and 2) report the psychometric and practical characteristics of the tools used to measure work ability. Table 6 (Online resource 3) reports detailed information about the tools’ predictive validity.

## Discussion

This review aimed at identifying and assessing the questionnaires used to measure individual psychosocial factors predictive of RTW among workers with MSDs or CMDs. We thus detected the individual psychosocial factors predictive of RTW. To our knowledge, this is the first work examining all the individual psychosocial predictors of RTW. A comparable review has been conducted, but it considered the measurement tools for only one individual psychosocial predictor of RTW (i.e., RTW expectations) [11, 12]. Our review identified three individual psychosocial factors that consistently predicted RTW among workers with MSDs or CMDs, that is, RTW self-efficacy, RTW expectations, and work ability. These three factors were all strong predictors of RTW after MSDs. However, only RTW self-efficacy was a strong predictor of RTW after CMDs. RTW expectations was a moderate predictor of RTW after CMDs and work ability was a limited predictor. These same factors have been identified as predictors of RTW for other diseases as well [7].

The studies included in the review show that more longitudinal studies have been conducted among workers with MSDs than CMDs. RTW expectations and work ability have been extensively studied, with 23 and 11 studies, respectively. Altogether, the other factors have been investigated in ten studies, with work involvement, self-perceived connection between health and job, optimism, and pessimism considered in only one study. In summary, 30 questionnaires about the three individual psychosocial factors with at least a moderate level of evidence of predictivity were analyzed. Of these instruments, only eight were multi-item scales; the 22 remaining tools were single-item measures for which it was impossible to provide a summary evaluation. Of the eight multi-item scales, only one was evaluated as questionable and five were evaluated as excellent.

### RTW Self-efficacy

Self-efficacy is a very well-known and studied construct in the psychological field. This longstanding tradition is reflected in the high quality of the measurement tools analyzed in this review. Given the strong theoretical foundation of the construct and the quality of the instrument available, more studies should investigate the role of RTW self-efficacy in the RTW process. We retrieved only seven studies (two with the same sample) investigating RTW self-efficacy as a predictor of RTW among workers with MSDs or CMDs, a relatively low number compared to the 24 studies retrieved for RTW expectations.

Among the five questionnaires retrieved in this review that are used to measure RTW self-efficacy, four were evaluated as excellent. The Return-to-Work Self-Efficacy Scale [38, 39] was characterized by a balance in the number of practical and psychometric criteria met by the tool. However, if one is more interested in the psychometric properties, the Return-to-Work Self-Efficacy Questionnaire [40–45] and the Return-to-Work Self-Efficacy Scale-19 [46] met all the psychometric criteria. It is worth noting that these last two scales share eight items. Alternatively, the Return-to-Work Obstacles and Self-Efficacy Scale (ROSES) [47] assesses, on 10 conceptual dimensions, potential RTW barriers perceived by workers (46 items), and then measures the self-efficacy in overcoming them. This questionnaire is especially suitable for clinical purposes to evaluate more salient barriers such as difficult relationships with RTW stakeholders (e.g., manager, colleagues) or apprehensions regarding cognitive difficulties.

### RTW Expectations

RTW expectations is the most studied factors among the four identified. This abundance of studies is partly due to how this factor is measured. Nineteen single-item measures were used, and only one scale. All these single items provided some predictive validity, as shown in Table 6 (Online resource 3), and they are short and easy to administer; therefore, RTW expectations can be measured in virtually every study at no cost. While this promotes extensive study of the factor and facilitates its evaluation in the clinical setting, we believe it also increases the risk of “HARKing”, in its form called “Suppress Loser Hypothesis” [56]. That is, the hypothesis of a significant effect of RTW expectations is not reported when results falsify it.

Moreover, none of the studies evaluated the reliability of the single-item measures. Even if it is commonly believed that single-item reliability cannot be estimated, this is not necessarily true [57, 58] and in fact should be estimated for RTW expectations, given the widespread adoption of single-item measures. However, estimating single-item reliability requires the presence of a validated scale consisting of more than one item. This step should be the first to be followed for the “RTW expectations” factor because the only scale proposed (i.e., Work-Related Recovery Expectations Questionnaire [13–15]) had some psychometric limitations, as shown in Table 4 (Online resource 1). A detailed discussion about the formulation of single items used to measure RTW expectations can be found in a dedicated review [59].

### Work Ability

In studies examining RTW from a psychosocial perspective, work ability is consistently defined as the worker’s evaluation of his/her personal work capability in light of his/her health condition and the work demands. However, having examined the entire body of scientific literature, it is evident that the concept of work ability has several different meanings [60]. This plurality of meanings explains why some readers may be disoriented by the adopted definition of work ability. A systematic scoping review analyzed this and the other definitions of work ability [60].

The ambiguous nature of the concept *work ability* is reflected, to some extent, in the two scales used to measure it that we retrieved in this review, the Work Ability Index and the Graded Reduced Work Ability Scale. The Work Ability Index, evaluated in this review as an excellent questionnaire, is very popular, especially in Europe. It was developed by members of the Finnish Institute of Occupational Health (FIOH) [50] and translated into more than 20 languages. The tool was supposed to be unidimensional, but data from different countries supported a factor solution with two dimensions [51]. Radkiewicz et al. [51] defined these two factors as the “objective” and “subjective” components of work ability. Commenting further on their results, we suggest that the two factors are the consequence of the mixing of two different conceptualizations of work ability. The “objective” factor reflects a biomedical conceptualization in which the physical impairments/diseases linearly determine work (dis)ability. The “subjective” factor closely reflects the definition of work ability that we adopted, i.e., work ability as the result of the interaction between the individual’s mental and physical health and the work demands [60]. This situation is not ideal since the Work Ability Index has only one final score. By considering the scores for the two factors separately, it would be more apparent which one of the two definitions is more useful in different contexts.

In this sense, the Single Item WAI question [32, 52–54] uses only one item of the Work Ability Index, the one with the highest factor loading on the “subjective” factor; it is, therefore, a precise measure of the “subjective” conceptualization of work ability. Moreover, the single item WAI question is the only single-item measure among all those retrieved in this review that has been validated. This validation was obtained by comparing performance on the single item with that on the full version of the Work Ability Index and represents a procedure that should be adopted more often when using single-item measures.

The Graded Reduced Work Ability Scale, evaluated in this review as a good measurement tool, presented a one-factor structure, but no factor loadings were reported in the article, and the variance explained by the single factor was 51% of the total variance [61]. Even in this scale, different conceptualizations of work ability are apparent if one examines the items. Beyond the personal evaluation of work capability in light of the health condition and the work demands, the Graded Reduced Work Ability Scale has items measuring other dimensions. These dimensions are the perceived work ability to perform “other work” (item 1), the perceived functional limitation due to the health complaint (item 3), the perceived severity of the health complaint (item 4), the perceived effect of the work activity on health (item 5), and a generic evaluation of other health complaints (item 6). It is worth noting that the predictive validity of the Graded Reduced Work Ability Scale was tested in only one study [61] but with discriminant analysis. The other study using the Graded Reduced Work Ability Scale [49] analyzed only three of the six items (reduced ability to work, the belief that work will aggravate the condition, and other complaints) as single items.

The Work Ability Index and the Graded Reduced Work Ability Scale were designed for practical purposes. These scales try to capture many facets of work ability in order to be as predictive as possible. This legitimate approach leaves room for psychometric improvements to be made to both scales.

## Strengths and Limitations

Other reviews have already investigated the measurement of RTW [62, 63], but this review is the first to examine the individual psychosocial predictors of RTW. The primary aim of the review was to identify and evaluate the questionnaires used to measure individual psychosocial factors predictive of RTW among workers with MSDs or CMDs. Pursuing this specific aim had two significant consequences. First, it was necessary to identify the predictive factors of RTW even though this was not the primary aim. For this reason, we did not focus our efforts on a meta-analysis of the effects of all the retrieved factors, which would have been the most reliable method for identifying the significant factors. Instead, we relied on a more resource-efficient approach: the “best-evidence synthesis procedure” [11]. We counted the significant and non-significant effects retrieved and determined the predictivity of the factor if the ratio between significant and non-significant effects was higher than a coefficient chosen a priori. While this may be a limitation of the present review, the procedure has already been successfully adopted in other reviews [6, 7, 64], and it is appropriate, given the primary aim of the review.

Second, substantial evidence in support of predictivity can be generated only when the predictors temporally precede the outcome. Therefore, we limited our inclusion criteria to prospective cohort studies. This criterion limited the number of studies and measurement tools we considered for all the individual psychosocial factors because many have been studied only in cross-sectional studies. More longitudinal studies are needed for individual psychosocial factors predictive of RTW among workers with CMDs. Other measurement tools with good psychometric and practical properties may exist but, because their predictive validity has not been tested longitudinally, they were not included in this review. While this may be considered a limitation of the present review, it also providesgreater confidence that the selected tools have predictive ability.

Another limitation of this review is the restriction on the type of psychosocial predictors and RTW outcomes we considered. As explained in the introduction, we focused on individual psychosocial factors related to the perception of the personal condition and motivation to RTW. Thus, other important psychosocial factors were not considered. Nevertheless, we acknowledge the relevance of age and gender because in phase 1—*Identification of the individual psychosocial factors*, we required the included studies to control for these two variables. Regarding the RTW outcomes, we considered the probability of being back at work at the time of study follow-up and the time to return to the workplace. There are other outcomes of the RTW process that have been used in the literature (e.g., number of days of absence during the observation time [65]). The studies that used these other RTW outcomes were not included in this review. Therefore, other sound measurement tools may not be included in this review because they were tested only against other RTW outcomes. Regardless, we believe that we identified most of the measurement tools used in the RTW literature, as the two definitions of RTW outcome we chose are those most frequently adopted in the studies investigating RTW specifically [63, 66].

## Conclusions

Promoting RTW after the onset of physical or mental disability has become crucial for the economy, society, and life of people in all industrialized countries. Despite the traditional importance of medical factors in the RTW process, individual psychosocial factors have been increasingly studied and considered crucial to the process. Today, it is recognized that these factors should be considered during the early phases of the RTW process. Our review provided a classification of the tools measuring individual psychosocial factors that have been used in the scientific literature and showing predictive validity among workers with MSDs and CMDs. The psychometric and practical characteristics of the measurement tools were identified, reported, and discussed in this study. We also proposed suggestions for improving the measurement of all the significant predictive factors based on the identified limitations of the measurement tools available. The list of measurement tools proposed can promote the use of high-quality existing instruments in new studies rather than the often-adopted practice of creating new questionnaires from scratch. Similarly, having a reference list of measurement tools can support the translation of high-quality instruments into new languages and their validation in new cultures.

Finally, we believe that the review results will be useful and valuable not only for researchers and clinicians working on work disability, but also for policymakers involved in developing RTW policies.

## Electronic supplementary material

Below is the link to the electronic supplementary material.

Supplementary file1 (PDF 230 kb)

Supplementary file2 (PDF 319 kb)

Supplementary file3 (PDF 389 kb)
