# Supplementary material for: A Systematic Search and Review of Questionnaires Measuring Individual psychosocial Factors Predicting Return to Work After Musculoskeletal and Common Mental Disorders
Source: J Occup Rehabil. 2020 Dec 23;31(3):491–511. doi: 10.1007/s10926-020-09935-6 (PMC8298352; doi:10.1007/s10926-020-09935-6)
Supplement: Supplementary file 1 — Supplementary file1 (PDF 230 kb) [file 10926_2020_9935_MOESM1_ESM.pdf]

Table 4 Psychometric evaluation of the Questionnaires

Questionnaires	Face validity	Construct validity	Convergent validity	Predictive validity	Internal consistency reliability	Test-retest reliability	Evaluation
**RTW expectation**							
Work-Related Recovery Expectations Questionnaire [1, 2]	High	-	-	√ MSD	$0.41 < \alpha < 0.75$ If first item removed $\alpha > .70$ (The last 2 items relate to future return-to-work whereas the first deals with perceived ability to return-to-work immediately)	-	2/6
**RTW Self-Efficacy**							
Self-efficacy for return to work questionnaire [3, 4] Appendix of Dionne et al. [5]	High	-	Correlation with: internal health locus of control = 0.04; powerful others health locus of control = -0.11**; chance health locus of control = -0.01; fear-avoidance beliefs-activity = -0.31**; fear-avoidance beliefs-work = -0.38**; average pain intensity of the past 6 months = -0.19**	√ MSD	$\alpha = .88$	-	4/6
Return-to-Work Self-Efficacy Scale	High	EFA and CFA	evaluated with readiness to RTW levels, RTW	√ MSD	Supervisor RTWSE α = 0.88-0.93; Pain RTWSE α = 0.76; Co-workers RTWSE α	-	5/6

[6, 7]			status, supervisor interaction, current pain, depression, health status		= 0.66-0.79; Overall self-efficacy scores α = 0.76		
Return-to-Work Self-Efficacy Scale-19 [8]	High	EFA	Correlation with: Pain intensity =-0.17**; Function = -0.31**; Activity avoidance = -0.19**; Physical demands = -0.09; Pain catastrophizing = - 0.10	√ MSD	Meeting job demands RTWSE α = .0.98; Modifying job tasks RTWSE α = 0.92; Communicating needs to others RTWSE α = 0.81	One week test-retest Pearson's r = .51 - .70	6/6
Return-to-work self-efficacy questionnaire [9]	High	EFA	Correlation with: General self-efficacy = .48** (N=88); Locus of control = .35** (N=91); Physical workload = .03 (N=1931); Depression = -.51** (N=1895); Active coping = .18** (N=1914); Avoidant coping = -.27** (N=1902)	√ CMD	α = .90	Two weeks test retest Pearson's r = .73**	6/6
Return-to-Work Obstacles and Self-Efficacy Scale [10]	High	EFA and CFA	-	√ MSD and CMD	α > .70 for all the dimension (MSD and CMD) except for Difficult relations with the insurance company (CMD) α = .64 and Difficult work/life balance α= .62	Two weeks test retest Pearson correlation of all factors	5/6

					(CMD) and α = .63 (MSD)	between .72** and 0.91**	
**Work Ability**							
Graded reduced work ability scale [11, 12]	High	EFA	-	√ MSD	α = .71 - .73	-	4/6
Work Ability Index [13–16]	High	EFA but a two-factorial solution performed better than the hypothesized single-factorial solution	Correlation with (N = 38000): General Health Index = 0.62; Burnout = -0.54; van Korff disability index = -0.52	√ MSD and CMD	α > .70 in six out of nine countries (0.54 – 0.79)	Four week test retes. The same WAI score in 25% of the subjects. Differences < 2 SD in 95% of the cases.	5/6
The single-item WAI question [17]	High	Ahlstrom et al. [17] compared the full version of the scale with the single item. Results suggest the single item may be a good alternative to the full scale.					

* p < .05; ** p < .01

References

1. Gross DP, Battié MC (2010) Recovery expectations predict recovery in workers with back pain but not other musculoskeletal conditions. J Spinal Disord Tech 23:451–456. https://doi.org/10.1097/BSD.0b013e3181d1e633

2. Gross DP, Battié MC (2005) Factors influencing results of functional capacity evaluations in workers' compensation claimants with low back pain. Phys Ther 85:315–22

3. Richard S, Dionne CE, Nouwen A (2011) Self-Efficacy and Health Locus of Control: Relationship to Occupational Disability Among Workers with Back Pain. J Occup Rehabil 21:421–430. https://doi.org/10.1007/s10926-011-9285-5

4. Dionne CE, Bourbonnais R, Frémont P, et al (2007) Determinants of "return to work in good health" among workers with back pain who consult in primary care settings: a 2-year prospective study. Eur Spine J 16:641–655. https://doi.org/10.1007/s00586-006-0180-2

5. Dionne CE, Bourbonnais R, Fremont P, et al (2005) A clinical return-to-work rule for patients with back pain. Can Med Assoc J 172:1559–1567. https://doi.org/10.1503/cmaj.1041159

6. Brouwer S, Amick BC, Lee H, et al (2015) The Predictive Validity of the Return-to-Work Self-Efficacy Scale for Return-to-Work Outcomes in Claimants with Musculoskeletal Disorders. J Occup Rehabil 25:725–732. https://doi.org/10.1007/s10926-015-9580-7

7. Brouwer S, Franche R-L, Hogg-Johnson S, et al (2011) Return-to-Work Self-Efficacy: Development and Validation of a Scale in Claimants with Musculoskeletal Disorders. J Occup Rehabil 21:244–258. https://doi.org/10.1007/s10926-010-9262-4

8. Shaw WS, Reme SE, Linton SJ, et al (2011) 3rd place, PREMUS best paper competition: development of the return-to-work self-efficacy (RTWSE-19) questionnaire – psychometric properties and predictive validity. Scand J Work Environ Health 37:109–119. https://doi.org/10.5271/sjweh.3139

9. Lagerveld SE, Blonk RWB, Brenninkmeijer V, Schaufeli WB (2010) Return to work among employees with mental health problems: Development and validation of a self-efficacy questionnaire. Work Stress 24:359–375. https://doi.org/http://dx.doi.org/10.1080/02678373.2010.532644

10. Corbière M, Negrini A, Durand M-J, et al (2017) Development of the Return-to-Work Obstacles and Self-Efficacy Scale (ROSES) and Validation with Workers Suffering from a Common Mental Disorder or Musculoskeletal Disorder. J Occup Rehabil 27:329–341. https://doi.org/10.1007/s10926-016-9661-2

11. Haldorsen EMH, Indahl A, Ursin H (1998) Patients with low back pain not returning to work - A 12-month follow-up study. Spine (Phila Pa 1976) 23:1202–1207. https://doi.org/10.1097/00007632-199806010-00004

12. Hagen EM, Svensen E, Eriksen HR (2005) Predictors and modifiers of treatment effect influencing sick leave in subacute low back pain patients. Spine (Phila Pa 1976) 30:2717–2723. https://doi.org/10.1097/01.brs.0000190394.05359.c7

13. Jensen AGC (2013) A two-year follow-up on a program theory of return to work intervention. Work 44:165–175. https://doi.org/10.3233/WOR-121497

14. Ekberg K, Wahlin C, Persson J, et al (2015) Early and Late Return to Work After Sick Leave: Predictors in a Cohort of Sick-Listed Individuals with Common Mental Disorders. J Occup Rehabil 25:627–637. https://doi.org/10.1007/s10926-015-9570-9

15. de Zwart BCH, Frings-Dresen MHW, van Duivenbooden JC (2002) Test-retest reliability of the Work Ability Index questionnaire. Occup Med (Lond) 52:177–81

16. Radkiewicz P, Widerszal-Bazyl M (2005) Psychometric properties of Work Ability Index in the light of comparative survey study. Int Congr Ser 1280:304–309. https://doi.org/10.1016/j.ics.2005.02.089

17. Ahlstrom L, Grimby-Ekman A, Hagberg M, Dellve L (2010) The work ability index and single-item question: Associations with sick leave, symptoms, and health - A prospective study of women on long-term sick leave. Scand J Work Environ Heal 36:404–412. https://doi.org/10.5271/sjweh.2917
