# Supplementary material for: A Systematic Search and Review of Questionnaires Measuring Individual psychosocial Factors Predicting Return to Work After Musculoskeletal and Common Mental Disorders
Source: J Occup Rehabil. 2020 Dec 23;31(3):491–511. doi: 10.1007/s10926-020-09935-6 (PMC8298352; doi:10.1007/s10926-020-09935-6)
Supplement: Supplementary file 2 — Supplementary file2 (PDF 319 kb) [file 10926_2020_9935_MOESM2_ESM.pdf]

Table 5 Practical evaluation of the Questionnaires

	Length	Final score computation	Cost	English version	Evaluation
RTW expectation					
Work-Related Recovery Expectations Questionnaire [1, 2]	Short - 3 items	Summative score but one item has to be reversed	Free	Original language: English	3/4
To what extent do you think you will return to work? [3]	Short – single item	None, easy	Free	Original language: Norwegian. Item reported in English	4/4
Do you expect to be able to return to your workplace? [4]	Short – single item	None, easy	Free	Original language: Danish. Item reported in English	4/4
"When do you think you will be able to work fulltime again." [5]	Short – single item	None, easy	Free	Original language: Dutch. Item reported in English	4/4
What is your opinion about your work ability in the long-term? [6]	Short – single item	None, easy	Free	Original language: Swedish. Item reported in English	4/4
What do you believe, honestly, is the probability that you will become so much better that you will be able to work at some time in the future? [7]	Short – single item	None, easy	Free	Original language: Swedish. Item reported in English	4/4
For how long do you believe you will be sick listed from today? [8]	Short – single item	None, easy	Free	Original language: Norwegian. Item reported in English	4/4
When do you think you will be able to work full-time again? [9]	Short – single item	None, easy	Free	Original language: Dutch. Item reported in English	4/4
Approximately how long do you think you will need to return to the job you	Short – single item	None, easy	Free	Original language: Spanish. Item reported in English	4/4

had before you went on sick leave? [10]					
How many months do you think it will take you to fully return to work? [11]	Short – single item	None, easy	Free	Original language: Dutch. Item reported in English	4/4
Expected duration of sick leave. [12]	Short – single item	None, easy	Free	Original language: Dutch. Item reported in English	4/4
How likely is it that within the next month you will have resumed some form of employment? [13]	Short – single item	None, easy	Free	Original language: French and English	4/4
Do you think you will be back to your normal work within 3 months? [14]	Short – single item	None, easy	Free	Original language: French. Item reported in English	4/4
Participants rated their certainty they would be working in 6 months [15, 16]	Short – single item	None, easy	Free, but exact wording of the question not reported	Original language: English and Spanish	3/4
Do you expect to return work within 6 months? [17]	Short – single item	None, easy	Free	Original language: Dutch and French. Item reported in English	4/4
In your estimation, what are the chances that you will be able to resume work in 6 months? [18]	Short – single item	None, easy	Free	Original language: Dutch and French. Item reported in English	4/4
In your estimation, what are the chances that you will be working in 6 months? [19]	Short – single item	None, easy	Free	Original language: Swedish. Item reported in English	4/4
What do you believe your situation concerning certified sickness absence will be in 4 weeks? [20]	Short – single item	None, easy	Free	Original language: Norwegian. Item reported in English	4/4

Expectations regarding RTW were measured by asking whether they expected to return to work within the next few weeks or not. [21]	Short – single item	None, easy	Free, but exact wording of the question not reported	Original language: Norwegian	3/4
I expect to be back at work within the next few weeks [22]	Short – single item	None, easy	Free	Original language: Norwegian. Item reported in English	4/4
RTW Self-Efficacy					
Self-efficacy for return to work questionnaire [23, 24]	Long – 8 items	Not defined – should give a final score between 0 and 100	Free	Original language: French. Item reported in English	2/4
Return-to-Work Self-Efficacy Scale [25, 26]	Long – 10 items	Summative scores or summative scores linearly transformed to range all between 2 and 10	Free	Original language: English	3/4
Return-to-Work Self-Efficacy Scale-19 [27]	Long – 19 items	Mean score over the 19 items	Free	Original language: English. There is a Danish validation [28]	2/4
Return-to-work self-efficacy questionnaire [29–31]	Long – 11 items	Mean score over the 11 items and 3 items has to be reversed	Free	Original language: Dutch. Item reported in English. There is a Brazilian validation [32]	2/4
Return-to-Work Obstacles and Self-Efficacy Scale [33]	Long – Only scales predictive of RTW: 25 items (x2). Full instrument 46 items (x2)	Mean score. Self-Efficacy (part B) measured only if the corresponding obstacle is perceived as potentially relevant (part A)	Free	Original language: French and English	2/4

Work Ability

Graded reduced work ability scale [34, 35]	Short – 6 items<br><br>or 3 items version [35]	Not specified [34] or item considered separately, dichotomized using the median score as<br><br>the split point [35]	Free	Original language: Norwegian. Item reported in English	3/4
Work Ability Index [36–39]	Long – 10 items	Scores have to be recoded and summed following the guidebook [40]	Free	Original language: Finish. Item reported in English. Translated versions with psychometric analyses does exist for other languages	2/4
The single-item WAI question [19, 41–43]	Short – single item	None, easy	Free	Same as the Work Ability Index	4/4
To what degree does your back disorder reduce your ability to perform your ordinary work today? [20]	Short – single item	None, easy	Free	Original language: Norwegian. Item reported in English	4/4
To what extent do you feel that, at this current moment in time, your ability to perform your usual job is lower than before? [10]	Short – single item	None, easy	Free	Original language: Norwegian. Item reported in English and used also in Spanish	4/4

References

1. Gross DP, Battié MC (2010) Recovery expectations predict recovery in workers with back pain but not other musculoskeletal conditions. J Spinal Disord Tech 23:451–456. https://doi.org/10.1097/BSD.0b013e3181d1e633

2. Gross DP, Battié MC (2005) Factors influencing results of functional capacity evaluations in workers' compensation claimants with low back pain. Phys Ther 85:315–22

3. Opsahl J, Eriksen HR, Tveito TH (2016) Do expectancies of return to work and Job satisfaction predict actual return to work in workers with long lasting LBP? BMC Musculoskelet Disord 17:481. https://doi.org/10.1186/s12891-016-1314-2

4. Nielsen MBD, Madsen IEH, Bultmann U, et al (2011) Predictors of return to work in employees sick-listed with mental health problems: findings from a longitudinal study. Eur J Public Health 21:806–811. https://doi.org/10.1093/eurpub/ckq171

5. Heymans MW, de Vet HCW, Knol DL, et al (2006) Workers' beliefs and expectations affect return to work over 12 months. J Occup Rehabil 16:685–695. https://doi.org/10.1007/s10926-006-9058-8

6. Heijbel B, Josephson M, Jensen I, et al (2006) Return to work expectation predicts work in chronic musculoskeletal and behavioral health disorders: Prospective study with clinical implications. J Occup Rehabil 16:173–184. https://doi.org/10.1007/s10926-006-9016-5

7. Lindell O, Johansson S-E, Strender L-E (2010) Predictors of stable return-to-work in non-acute, non-specific spinal pain: low total prior sick-listing, high self prediction and young age. A two-year prospective cohort study. BMC Fam Pract 11:53. https://doi.org/10.1186/1471-2296-11-53

8. Aasdahl L, Pape K, Jensen C, et al (2018) Associations Between the Readiness for Return to Work Scale and Return to Work: A Prospective Study. J Occup Rehabil 28:97–106. https://doi.org/10.1007/s10926-017-9705-2

9. Richter J, Blatter B, Heinrich J, et al (2011) Prognostic factors for disability claim duration due to musculoskeletal symptoms among self-employed persons. BMC Public Health 11:945. https://doi.org/10.1186/1471-2458-11-945

10. Sampere M, Gimeno D, Serra C, et al (2012) Return to Work Expectations of Workers on Long-Term Non-Work-Related Sick Leave. J Occup Rehabil 22:15–26. https://doi.org/10.1007/s10926-011-9313-5

11. Nieuwenhuijsen K, Verbeek JH, de Boer AG, et al (2006) Predicting the duration of sickness absence for patients with common mental disorders in occupational health care. Scand J Work Environ Health 32:67–74. https://doi.org/10.5271/sjweh.978

12. Steenstra IA, Koopman FS, Knol DL, et al (2005) Prognostic factors for duration of sick leave due to low-back pain in dutch health care professionals. J Occup Rehabil 15:591–605. https://doi.org/10.1007/s10926-005-8037-9

13. Carriere JS, Thibault P, Sullivan MJL (2015) The Mediating Role of Recovery Expectancies on the Relation Between Depression and Return-to-Work. J Occup Rehabil 25:348–356. https://doi.org/10.1007/s10926-014-9543-4

14. Dionne CE, Bourbonnais R, Fremont P, et al (2005) A clinical return-to-work rule for patients with back pain. Can Med Assoc J 172:1559–1567. https://doi.org/10.1503/cmaj.1041159

15. Turner JA, Franklin G, Fulton-Kehoe D, et al (2006) Worker recovery expectations and fear-avoidance predict work disability in a population-based workers' compensation back pain sample. Spine (Phila Pa 1976) 31:682–689. https://doi.org/10.1097/01.brs.0000202762.88787.af

16. Turner JA, Franklin G, Fulton-Kehoe D, et al (2007) Early predictors of chronic work disability

associated with carpal tunnel syndrome: A longitudinal workers' compensation cohort study. Am J Ind Med 50:489–500. https://doi.org/10.1002/ajim.20477

17. Du Bois M, Szpalski M, Donceel P (2009) Patients at risk for long-term sick leave because of low back pain. Spine J 9:350–359. https://doi.org/10.1016/j.spinee.2008.07.003

18. Du Bois M, Donceel P (2008) A screening questionnaire to predict no return to work within 3 months for low back pain claimants. Eur Spine J 17:380–385. https://doi.org/10.1007/s00586-007-0567-8

19. Wåhlin C, Ekberg K, Persson J, et al (2012) Association between clinical and work-related interventions and return-to-work for patients with musculoskeletal or mental disorders. J Rehabil Med 44:355–62. https://doi.org/10.2340/16501977-0951

20. Reiso H, F Nygård J, S Jørgensen G, et al (2003) Back to Work: Predictors of Return to Work Among Patients With Back Disorders Certified As Sick. Spine (Phila Pa 1976) 28:1468–1473. https://doi.org/10.1097/01.BRS.0000067089.83472.1F

21. Reme SE, Hagen EM, Eriksen HR (2009) Expectations, perceptions, and physiotherapy predict prolonged sick leave in subacute low back pain. BMC Musculoskelet Disord 10:139. https://doi.org/10.1186/1471-2474-10-139

22. Løvvik C, Shaw W, Øverland S, Reme SE (2014) Expectations and illness perceptions as predictors of benefit recipiency among workers with common mental disorders: Secondary analysis from a randomised controlled trial. BMJ Open 4:1–9. https://doi.org/10.1136/bmjopen-2013-004321

23. Richard S, Dionne CE, Nouwen A (2011) Self-Efficacy and Health Locus of Control: Relationship to Occupational Disability Among Workers with Back Pain. J Occup Rehabil 21:421–430. https://doi.org/10.1007/s10926-011-9285-5

24. Dionne CE, Bourbonnais R, Frémont P, et al (2007) Determinants of "return to work in good health" among workers with back pain who consult in primary care settings: a 2-year prospective study. Eur Spine J 16:641–655. https://doi.org/10.1007/s00586-006-0180-2

25. Brouwer S, Amick BC, Lee H, et al (2015) The Predictive Validity of the Return-to-Work Self-Efficacy Scale for Return-to-Work Outcomes in Claimants with Musculoskeletal Disorders. J Occup Rehabil 25:725–732. https://doi.org/10.1007/s10926-015-9580-7

26. Brouwer S, Franche R-L, Hogg-Johnson S, et al (2011) Return-to-Work Self-Efficacy: Development and Validation of a Scale in Claimants with Musculoskeletal Disorders. J Occup Rehabil 21:244–258. https://doi.org/10.1007/s10926-010-9262-4

27. Shaw WS, Reme SE, Linton SJ, et al (2011) 3rd place, PREMUS best paper competition: development of the return-to-work self-efficacy (RTWSE-19) questionnaire – psychometric properties and predictive validity. Scand J Work Environ Health 37:109–119. https://doi.org/10.5271/sjweh.3139

28. Momsen AMH, Rosbjerg R, Stapelfeldt CM, et al (2016) Cross-cultural adaptation and validation of the danish version of the 19-item return-to-work self-efficacy (RTWSE-19) questionnaire. Scand J Work Environ Heal 42:338–345. https://doi.org/10.5271/sjweh.3568

29. Lagerveld SE, Blonk RWB, Brenninkmeijer V, Schaufeli WB (2010) Return to work among employees with mental health problems: Development and validation of a self-efficacy questionnaire. Work Stress 24:359–375. https://doi.org/http://dx.doi.org/10.1080/02678373.2010.532644

30. Lagerveld SE, Brenninkmeijer V, Blonk RWBB, et al (2017) Predictive value of work-related self-efficacy change on RTW for employees with common mental disorders. Occup Environ Med 74:381–383. https://doi.org/10.1136/oemed-2016-104039

31. Nieuwenhuijsen K, Noordik E, van Dijk FJH, van der Klink JJ (2013) Return to Work Perceptions and Actual Return to Work in Workers with Common Mental Disorders. J Occup Rehabil 23:290–299.

https://doi.org/10.1007/s10926-012-9389-6

32. Silva-Junior JS, Griep RH, Lagerveld SE, Fischer FM (2017) Brazilian cross-cultural adaptation of ``Return-to-work self-efficacy{''} questionnaire. Rev Saude Publica 51:. https://doi.org/10.1590/S1518-8787.2017051006778

33. Corbière M, Negrini A, Durand M-J, et al (2017) Development of the Return-to-Work Obstacles and Self-Efficacy Scale (ROSES) and Validation with Workers Suffering from a Common Mental Disorder or Musculoskeletal Disorder. J Occup Rehabil 27:329–341. https://doi.org/10.1007/s10926-016-9661-2

34. Haldorsen EMH, Indahl A, Ursin H (1998) Patients with low back pain not returning to work - A 12-month follow-up study. Spine (Phila Pa 1976) 23:1202–1207. https://doi.org/10.1097/00007632-199806010-00004

35. Hagen EM, Svensen E, Eriksen HR (2005) Predictors and modifiers of treatment effect influencing sick leave in subacute low back pain patients. Spine (Phila Pa 1976) 30:2717–2723. https://doi.org/10.1097/01.brs.0000190394.05359.c7

36. Jensen AGC (2013) A two-year follow-up on a program theory of return to work intervention. Work 44:165–175. https://doi.org/10.3233/WOR-121497

37. Ekberg K, Wahlin C, Persson J, et al (2015) Early and Late Return to Work After Sick Leave: Predictors in a Cohort of Sick-Listed Individuals with Common Mental Disorders. J Occup Rehabil 25:627–637. https://doi.org/10.1007/s10926-015-9570-9

38. de Zwart BCH, Frings-Dresen MHW, van Duivenbooden JC (2002) Test-retest reliability of the Work Ability Index questionnaire. Occup Med (Lond) 52:177–81

39. Radkiewicz P, Widerszal-Bazyl M (2005) Psychometric properties of Work Ability Index in the light of comparative survey study. Int Congr Ser 1280:304–309. https://doi.org/10.1016/j.ics.2005.02.089

40. Tuomi K, Ilmarinen J, Jahkola A, Katajarinnel TA (1998) Work Ability Index. Occupational Health Care

41. Netterstrøm B, Eller NH, Borritz M (2015) Prognostic Factors of Returning to Work after Sick Leave due to Work-Related Common Mental Disorders: A One- and Three-Year Follow-Up Study. Biomed Res Int 2015:1–7. https://doi.org/10.1155/2015/596572

42. Kuijer PPFM, Gouttebarge V, Wind H, et al (2012) Prognostic value of self-reported work ability and performance-based lifting tests for sustainable return to work among construction workers. Scand J Work Environ Heal 38:600–603. https://doi.org/10.5271/sjweh.3302

43. van der Giezen AM, Bouter LM, Nijhuis FJN (2000) Prediction of return-to-work of low back pain patients sicklisted for 3-4 months. Pain 87:285–294. https://doi.org/10.1016/S0304-3959(00)00292-X
