# Supplementary material for: A Systematic Search and Review of Questionnaires Measuring Individual psychosocial Factors Predicting Return to Work After Musculoskeletal and Common Mental Disorders
Source: J Occup Rehabil. 2020 Dec 23;31(3):491–511. doi: 10.1007/s10926-020-09935-6 (PMC8298352; doi:10.1007/s10926-020-09935-6)
Supplement: Supplementary file 3 — Supplementary file3 (PDF 389 kb) [file 10926_2020_9935_MOESM3_ESM.pdf]

Table 6 Tools Predictive validity

	Reference	Outcome	Sample	Follow Up	Analysis	Crude Effect	Adjusted Effect
RTW expectation							
Work-Related Recovery Expectations Questionnaire	[1]	Days until claim closure	N = 298, LBP	1 year	Cox reg.	Worst Expectation HR = 0.84 [0.74, 0.96]	Worst Expectation HR = 0.83 [0.73, 0.96]
			N = 742, MDS not LBP	1 year	Cox reg.	Worst Expectation HR = ns	Worst Expectation HR = ns
	[2]	Days until claim closure	N = 97, LBP	1 year	Cox reg.	Worst Expectation HR = 0.78 [0.65, 0.95]	Worst Expectation HR = 0.83 [0.73, 0.96]
To what extent do you think you will return to work?	[3]	RTW	N = 567, LBP	1 year	Log. reg.	Ref.: Low or moderate expectancies<br>Men,High OR = 5.38 [2.81, 10.33]<br>Women,High OR = 4.80 [2.47, 9.35]	Ref.: Low or moderate expectancies<br>Men,High OR = 4.17 [1.9, 9.17]<br>Women,High OR = 3.36 [1.58, 7.14]
Do you expect to be able to return to your workplace?	[4]	Weeks until RTW	N = 644, CMD	1 year	Cox reg.	RTW expectation (yes) HR = 1.35 [1.08, 1.77]	RTW expectation (yes) HR = 1.27 [1.01, 1.61]
When do you think you will be able to work fulltime again?	[5]	RTW for at least 4 weeks	N = 268, LBP	1 year	Cox reg.	Increased RTW expected time HR = 0.94 [0.90, 0.98]	HR = 0.95 [0.91– 1.00]
What is your opinion about your work ability in the long-term? [6]	[6]	RTW for at least 15 days	N = 355, MSD & CMD	1.5 year	Log. reg.	Positive prediction OR = 15.99 [6.86–37.25]	Positive prediction OR = 8.28 [3.31, 20.69]

What do you believe, honestly, is the probability that you will become so much better that you will be able to work at some time in the future?	[7]	RTW for at least 1 month	N = 122, MSD	0.5, 1, 1.5, and 2 years	Log. reg.	High self prediction OR (0.5 year) = 4.2 [1.2, 15.2]; OR (1 year) = 6.4 [1.9, 21]; OR (1.5 year) = 4.4 [1.5, 12.4]; OR (2 years) = 3.8 [1.4, 10.2]	High self prediction OR (0.5 year) = 4.1 [1.1, 15.7]; OR (1 year) = 5.2 [1.5, 17.5]; OR (1.5 year) = ns; OR (2 years) = 2.7 [0.9, 7.8]
For how long do you believe you will be sick listed from today?	[8]	RTW for at least 30 consecutive days	N = 85, MSD & CMD	9 months	Log. reg.	Not reported	less than 2 months OR = 0.65 [0.47 , 0.84]; 2,4 months OR = 0.14 [0.01 , 0.27]; 4,10 months OR = 0.18 [0.02 , 0.34]; more than 10 months OR = 0.08 [0.00 , 0.22]
When do you think you will be able to work full-time again? Richter et al.	[9]	Days of work disability compensation received until RTW	N = 243 self-employed, MSD	1 year	Cox. reg.	Ref.: RTW exp. < 1 month; >1 month HR = 0.28 "No idea" HR = 0.25	Ref.: RTW exp. < 1 month; >1 month HR = 0.24 [0.15, 0.38] "No idea" HR = 0.23 [0.15, 0.34]
Approximately how long do you think you will need to return to	[10]	Days of sick leave until	N = 314 MSD;	2 years	Cox. reg.		Ref.: RTW exp. < 1 month;

the job you had before you went on sick leave?		RTW				Available only for the two populations together.<br>Ref.: RTW exp. < 1 month;<br>1,3 months HR = 0.45 [0.36, 0.58];	1–3 months HR = 0.59 [0.42, 0.84],<br>>3 months HR = 0.36 [0.20, 0.64],<br>“Never” HR = 0.36 [0.09, 1.52],<br>Don’t know HR = 0.50 [0.36, 0.68];
			N = 119 CMD	2 years	Cox. reg.	>3 months HR = 0.32 [0.23, 0.46]<br>“Never” HR = 0.09 [0.04, 0.21];<br>Don’t know HR = 0.36 [0.29, 0.43]	Ref.: RTW exp. < 1 month;<br>1–3 months HR = 0.34 [0.16, 0.69],<br>>3 months HR = 0.41 [0.12, 1.35],<br>“Never” HR = 0.12 [0.03, 0.43],<br>Don’t know HR = 0.39 [0.23, 0.67]
How many months do you think it will take you to fully return to work?	[11]	Days until RTW with the same number of hours as before the sick leave for at least 1 week	N = 168, CMD	1 year	Cox reg.	Not reported	Recovery expectations > 3 months HR = 0.5 [0.3, 0.8]
Expected duration of	[12]	Days until RTW with	N = 514, LBP	6 months	Cox reg.	Expected RTW ≥ 10 days HR = 0.273	Expected RTW ≥ 10 days

sick leave.		equal earnings as before the sick absence for at least 4 weeks				[0.21, 0.36]	HR = 0.286 [0.19, 0.44]
How likely is it that within the next month you will have resumed some form of employment?	[13]	Full time RTW	N = 109, MSD	1 year	Log. reg.	Not reported	Low RTW expt. OR = 0.96 [0.94, 0.98]
Participants rated their certainty they would be working in 6 months	[14]	Wage replacement for work disability at FwUp yes=1	N = 1068, LBP	6 months	Log reg.	Ref.: Very high expect.<br>Very low OR = 9.18 [5, 16.84]<br>Low OR=6.45 [3.38, 12.30]<br>Moderate OR=3.35 [2.15, 5.22]<br>High OR=1.95 [1.18, 3.20]<br>Don't know/didn't answer OR=5.89 [3.16, 10.96]	Ref.: Very high expect.<br>Very low OR = 3.08 [1.46, 6.48]<br>Low OR = ns<br>Moderate OR= ns<br>High OR = ns<br>Don't know/didn't answer OR = 2.93 [1.36, 6.32]
Participants rated their certainty they would be working in 6 months	[15]	Wage replacement for work disability at FwUp yes=1	N = 899, CTS	6 months	Log reg.	Ref.: Very high expect. High OR = 1.90 [1.18, 3.08]<br>Moderate OR = 4.22	Ref.: Very high expect. High OR = ns<br>Moderate OR = 2.9 [1.7, 5.1]

						[2.61, 6.81]<br>Low OR = 5.87 [3.73, 9.25]<br>Did not answer OR = 5.09 [2.46, 10.52]	Low OR = 3.0 [1.8, 5.2]<br>Did not answer OR = 4.3 [1.6, 11.8]
Do you expect to return work within 6 months?	[16]	Non-RTW at Follow-up	N = 346, LBP	3 months	Log. reg.	Not reported	[Not clear how the response scale was used. Likely it was 0 *Very large chance* and 10 *No chance*]<br>OR = 1.14 [1.04, 1.25]
In your estimation, what are the chances that you will be able to resume work in 6 months?	[17]	Non-RTW at Follow-up	N = 186, LBP	3 months	Log. reg.	Ref.: Very sure to RTW within 6 months (equal to 10 on the Likert scale);<br>Not very sure to RTW (< 10 on the Likert scale) OR = 5.2 [2.4, 11.1]	Ref.: Very sure to RTW within 6 months [equal to 10 on the Likert scale];<br>Not very sure to RTW [< 10 on the Likert scale] OR = 4.6 [2.1, 10.3]
In your estimation, what are the chances that you will be working in 6 months?	[18]	RTW	N = 432, MSD;	3 months	Log. reg.	Not reported	High RTW exp.<br>OR = 1.21 [1.07, 1.38]
			N = 267, CMD	3 months	Log. reg	Not reported	High RTW exp. OR = 1.84 [0.73, 4.62]
What do you believe your situation concerning certified sickness absence will	[19]	RTW for<br>at least 60 consecutive days	N = 190, LBP	2 years	Cox reg.	HR not reported, $p < .01$	Ref.: RTW;<br>Continued certification HR = 0.31 [0.17, 0.54]

be in 4 weeks?							
Expectations regarding RTW were measured by asking whether they expected to return to work within the next few weeks or not.	[20]	Non-RTW at Follow-up	N = 176, LBP	3 months	Log. reg.	Not reported	None or negative expectations to RTW, OR = 4.2 [1.7, 10]
				1 year	Log. reg.	Not reported	None or negative expectations to RTW, OR = 1.9 [0.9, 4.0]
				2 years	Log. reg.	Not reported	None or negative expectations to RTW, OR = 2.0 [0.9, 4.3]
I expect to be back at work within the next few weeks	[21]	Non-RTW at Follow-up	N = 241, LBP	6 months	Log. reg.	Not reported	Ref.: Positive RTW expectations; Uncertain exp. OR = 2.62 [1.47, 4.67], Negative exp. OR= 3.78 [2.11, 6.76]
RTW Self-Efficacy							
Self-efficacy for return to work questionnaire	[22]	Not RTW and at least one attempt to RTW during follow-up	N = 588, MSD	2 years	Log. reg.	Ref: Low RTW SE; Moderate SE OR = 0.61 [0.37, 1]; High SE OR = 0.18 [0.09, 0.36]	Ref: Low RTW SE; Moderate SE OR = 0.82 [0.47, 1.40]; High SE OR = 0.28 [0.14, 0.57]
		Not RTW and no attempt to RTW	N = 551, MSD	2 years	Log. reg.	Ref: Low RTW SE; Moderate SE OR = 0.35 [0.19, 0.67];	Ref: Low RTW SE; Moderate SE OR = 0.51 [0.26, 1]; High SE OR =

						High SE OR = 0.11 [0.04, 0.27]	0.19 [0.07, 0.48]
Return-to-Work Self-Efficacy Scale	[23]	RTW	N = 419, MSD	6 months	Log. reg.	Overall score RTWSE OR = 1.21 [1.06, 1.38], Pain RTWSE OR = 1.26 [1.23, 1.41], other n.s.	Pain RTWSE OR=1.20 [1.06, 1.37], other n.s.
			N = 366, MSD	1 year	Log. reg.	Overall score RTWSE OR = 1.20 [1.04, 1.39], Pain RTWSE OR = 1.14 [1.01, 1.28], other n.s.	n.s.
Return-to-Work Self-Efficacy Scale-19	[24]	Full RTW: full duty	N = 292, LBP	3 months	Log. reg.	Ref.: Low RTWSE; medium RTWSE RR[c] = 1.54 [0.68, 3.46]; high RWSE RR[c] = 2.94 [1.27, 6.78]	Ref.: Low RTWSE; medium RTWSE RR[c] = 3.62 [0.70, 3.75]; high RWSE RR[c] = 3.72 [1.51, 9.13]
Return-to-work self-efficacy questionnaire	[25]	Full RTW: working the full contract hours	N = 245, CMD	3 months	Log. reg.	RTW-SE OR = 1.37**	RTW-SE OR = 1.40 [1.09, 1.79]
	[26]	Full RTW: working the full contract hours	N = 168, CMD	9 months	Cox. reg.	RTW-SE HR = 2.82 [2.03, 3.93]	RTW-SE HR = 3.16 [2.04, 4.87]

	[27][b]	Full RTW: working the full contract hours	N = 145, CMD	6 months	Kaplan–Meier survival curve	positive RTW-SE being associated with a shorter time until return to work [χ^2 = 17.8 [df = 1], P < .001]	Not tested
Return-to-Work Obstacles and Self-Efficacy Scale	[28]	RTW at the same employer's	N = 206, MSD	6 months	Log. reg.	Not reported	Job Demand SE OR = 1.62, [1.09, 2.42]; Fears of a relapse SE OR = 1.52 [1.13, 2.03]; Feeling of organizational injustice SE OR = 1.79 [1.12, 2.85]; Difficult relation with the immediate supervisor SE OR = 2.75 [1.45, 5.21]
			N = 157, CMD	6 months	Log. reg.	Not reported	Cognitive difficulties SE OR = 1.45, [1.04, 2.01]; Job demands SE OR = 1.59, [1.11, 2.28]
Work Ability							
Graded reduced work ability scale	[29]	No RTW	N = 260	1 year	Discriminant analysis	Reported reduced ability to continue in ordinary working [p = .04]	Combined with other medical, sociodemographic and psychological variable resulted in a classification accuracy of 77%
	[30]	No RTW	N = 457	3 months	Log. reg.	Very large reduced ability to regularly	Belief that work would aggravate the condition

						work OR = 2.9 [2, 4.4]; Believe work will aggravate condition OR = 2.9 [2, 4.4]; Other complaints that affect health OR = 1.7 [1.0, 2.7]	OR = 2.3 [1.3, 3.9]; Large reduced ability to regularly work OR = 0.8 [0.4, 1.6]; large reduced ability to regularly work*the control group (vs intervention group) OR = 2.8[1.0, 7.6]
				1 Year		Very large reduced ability to regularly work OR = 1.3 [0.9, 1.9]; Believe work will aggravate condition OR = 1.8 [1.2, 2.7]; Other complaints that affect health OR = 2.2 [1.4, 3.4]	Belief that work would aggravate the condition OR = 1.5 [1.0–2.5]
Work ability index (WAI)	[31]	Early RTW (< 3months),	N = 179, CMD	3 months	Cox reg.	Not reported	WAI HR = 1.08 [1.05, 1.10]
		Later RTW (3 – 12 moths)	N = 98, CMD	1 year	Cox reg.	Not reported	WAI HR = ns
	[32]	RTW	N = 87, MSD & CMD	1 years	Log. reg.	WAI OR = 5.4 [1.9, 15.4]	WAI OR = 5.7 [1.6, 20.1]
				2 years	Log. reg.	WAI OR = 4.1 [1.5,	WAI OR = 5.6 [2.1, 15.0]

						10.7]	
	[33]	RTW	N = 223, CMD	1 year	Log. reg.	*t* test, diff. In WAI mean between RTW and not RTW, p = .001	WAI OR = 1.24 [1.03, 1.40]
			N = 143, CMD	3 years	Log. reg.	*t* test, diff. In WAI mean between RTW and not RTW, p = .04	Results not shown, but similar to those at 1 year
The single-item WAI question	[34]	Days until sustained RTW (4 weeks)	N = 72, MSD	1 year	Linear reg.	b = -11.8 [-20.5, -3.1]	b = -11.7 [-20.2, -3.3]
	[18]	RTW	N = 432, MSD	3 months	Log. reg.	Not reported	WAI OR = 3.89 [1.24, 12.17]
			N = 267, CMD	3 months	Log. reg.	Not reported	WAI OR = 1.12 [0.93, 1.36]
	[35]	RTW	N = 298, LBP	2 years	Log. reg.	WAI OR =1.14 [1.01, 1.29]	ns
To what degree does your back disorder reduce your ability to perform your ordinary work today?	[19]	RTW for at least 60 consecutive days	N = 190, LBP	2 years	Cox reg.	HR not reported, p < .01	Ref.: "Moderately reduced". "Much red." HR = 0.63 [0.36, 1.10]; "Very much red." HR = 0.43 [0.25, 0.73]
To what extent do you feel that, at this	Sampere et al.	Days until RTW in the	N = 314, MSD	2 years	Cox reg.	Available only for the two populations	Moderately reduced HR = 0.92 [0.63, 1.34]

current moment in time, your ability to perform your usual job is lower than before? [10]	same company as before the sick leave				together: Ref.: "Not at all or slightly reduced". "Very or extremely reduced" HR = 0.47 [0.37–0.59]	Very or extremely reduced HR = 0.49 [0.33, 0.72]
		N = 119, CMD	2 years	Cox reg.		"Moderately reduced" HR = 4.14 [0.87–19.72]<br>"Very or extremely reduced" HR = 2.93 [0.62–13.92]

Note. CMD = common mental disorder; MSD = musculoskeletal disorder; LBP = low back pain

Ref. = the reference category of the predictor under consideration in the regression when this predictor is categorical

Log. reg. = Logistic regression; Cox reg. = Cox regression

It was not possible to list the effects founded in Dionne et al. [36] because they used Recursive partitioning. Therefore, the effect of RTW expectation in the article is in interaction with other variables whereas the studies in this table used regression procedure and presented the principal effects.

[a]The same sample is used in Schultz et al. [37] and in Schultz et al. [38]

[b] Nieuwenhuijsen et al. [27] was not included in the count of evidences for RTW Self-Efficacy because predictivity was tested only univariately

[c] Shaw et al., [24] reported the results as risk ratio (RR) but did not report any indication of how odd ratios (OR) were converted in RRs. Therefore, it is not clear if the results reported are RRs or ORs.

References

1. Gross DP, Battié MC (2010) Recovery expectations predict recovery in workers with back pain but not other musculoskeletal conditions. J Spinal Disord Tech 23:451–456. https://doi.org/10.1097/BSD.0b013e3181d1e633
2. Gross DP, Battié MC (2005) Work-related recovery expectations and the prognosis of chronic low back pain within a workers' compensation setting. J Occup Environ Med 47:428–433
3. Opsahl J, Eriksen HR, Tveito TH (2016) Do expectancies of return to work and Job satisfaction predict actual return to work in workers with long lasting LBP? BMC Musculoskelet Disord 17:481. https://doi.org/10.1186/s12891-016-1314-2
4. Nielsen MBD, Madsen IEH, Bultmann U, et al (2011) Predictors of return to work in employees sick-listed with mental health problems: findings from a longitudinal study. Eur J Public Health 21:806–811. https://doi.org/10.1093/eurpub/ckq171
5. Heymans MW, de Vet HCW, Knol DL, et al (2006) Workers' beliefs and expectations affect return to work over 12 months. J Occup Rehabil 16:685–695. https://doi.org/10.1007/s10926-006-9058-8
6. Heijbel B, Josephson M, Jensen I, et al (2006) Return to work expectation predicts work in chronic musculoskeletal and behavioral health disorders: Prospective study with clinical implications. J Occup Rehabil 16:173–184. https://doi.org/10.1007/s10926-006-9016-5
7. Lindell O, Johansson S-E, Strender L-E (2010) Predictors of stable return-to-work in non-acute, non-specific spinal pain: low total prior sick-listing, high self prediction and young age. A two-year prospective cohort study. BMC Fam Pract 11:53. https://doi.org/10.1186/1471-2296-11-53
8. Aasdahl L, Pape K, Jensen C, et al (2018) Associations Between the Readiness for Return to Work Scale and Return to Work: A Prospective Study. J Occup Rehabil 28:97–106. https://doi.org/10.1007/s10926-017-9705-2
9. Richter J, Blatter B, Heinrich J, et al (2011) Prognostic factors for disability claim duration due to musculoskeletal symptoms among self-employed persons. BMC Public Health 11:945. https://doi.org/10.1186/1471-2458-11-945
10. Sampere M, Gimeno D, Serra C, et al (2012) Return to Work Expectations of Workers on Long-Term Non-Work-Related Sick Leave. J Occup Rehabil 22:15–26. https://doi.org/10.1007/s10926-011-9313-5
11. Nieuwenhuijsen K, Verbeek JH, de Boer AG, et al (2006) Predicting the duration of sickness absence for patients with common mental disorders in occupational health care. Scand J Work Environ Health 32:67–74. https://doi.org/10.5271/sjweh.978
12. Steenstra IA, Koopman FS, Knol DL, et al (2005) Prognostic factors for duration of sick leave due to low-back pain in dutch health care professionals. J Occup Rehabil 15:591–605. https://doi.org/10.1007/s10926-005-8037-9
13. Carriere JS, Thibault P, Sullivan MJL (2015) The Mediating Role of Recovery Expectancies on the Relation Between Depression and Return-to-Work. J Occup Rehabil 25:348–356. https://doi.org/10.1007/s10926-014-9543-4

14. Turner JA, Franklin G, Fulton-Kehoe D, et al (2006) Worker recovery expectations and fear-avoidance predict work disability in a population-based workers' compensation back pain sample. Spine (Phila Pa 1976) 31:682–689. https://doi.org/10.1097/01.brs.0000202762.88787.af

15. Turner JA, Franklin G, Fulton-Kehoe D, et al (2007) Early predictors of chronic work disability associated with carpal tunnel syndrome: A longitudinal workers' compensation cohort study. Am J Ind Med 50:489–500. https://doi.org/10.1002/ajim.20477

16. Du Bois M, Szpalski M, Donceel P (2009) Patients at risk for long-term sick leave because of low back pain. Spine J 9:350–359. https://doi.org/10.1016/j.spinee.2008.07.003

17. Du Bois M, Donceel P (2008) A screening questionnaire to predict no return to work within 3 months for low back pain claimants. Eur Spine J 17:380–385. https://doi.org/10.1007/s00586-007-0567-8

18. Wåhlin C, Ekberg K, Persson J, et al (2012) Association between clinical and work-related interventions and return-to-work for patients with musculoskeletal or mental disorders. J Rehabil Med 44:355–62. https://doi.org/10.2340/16501977-0951

19. Reiso H, F Nygård J, S Jørgensen G, et al (2003) Back to Work: Predictors of Return to Work Among Patients With Back Disorders Certified As Sick. Spine (Phila Pa 1976) 28:1468–1473. https://doi.org/10.1097/01.BRS.0000067089.83472.1F

20. Reme SE, Hagen EM, Eriksen HR (2009) Expectations, perceptions, and physiotherapy predict prolonged sick leave in subacute low back pain. BMC Musculoskelet Disord 10:139. https://doi.org/10.1186/1471-2474-10-139

21. Løvvik C, Shaw W, Øverland S, Reme SE (2014) Expectations and illness perceptions as predictors of benefit recipiency among workers with common mental disorders: Secondary analysis from a randomised controlled trial. BMJ Open 4:1–9. https://doi.org/10.1136/bmjopen-2013-004321

22. Richard S, Dionne CE, Nouwen A (2011) Self-Efficacy and Health Locus of Control: Relationship to Occupational Disability Among Workers with Back Pain. J Occup Rehabil 21:421–430. https://doi.org/10.1007/s10926-011-9285-5

23. Brouwer S, Amick BC, Lee H, et al (2015) The Predictive Validity of the Return-to-Work Self-Efficacy Scale for Return-to-Work Outcomes in Claimants with Musculoskeletal Disorders. J Occup Rehabil 25:725–732. https://doi.org/10.1007/s10926-015-9580-7

24. Shaw WS, Reme SE, Linton SJ, et al (2011) 3rd place, PREMUS best paper competition: development of the return-to-work self-efficacy (RTWSE-19) questionnaire – psychometric properties and predictive validity. Scand J Work Environ Health 37:109–119. https://doi.org/10.5271/sjweh.3139

25. Lagerveld SE, Blonk RWB, Brenninkmeijer V, Schaufeli WB (2010) Return to work among employees with mental health problems: Development and validation of a self-efficacy questionnaire. Work Stress 24:359–375. https://doi.org/http://dx.doi.org/10.1080/02678373.2010.532644

26. Lagerveld SE, Brenninkmeijer V, Blonk RWBB, et al (2017) Predictive value of work-related self-efficacy change on RTW for employees with common mental disorders. Occup Environ Med 74:381–383. https://doi.org/10.1136/oemed-2016-104039

27. Nieuwenhuijsen K, Noordik E, van Dijk FJH, van der Klink JJ (2013) Return to Work Perceptions and Actual Return to Work in Workers with Common Mental Disorders. J Occup Rehabil 23:290–299. https://doi.org/10.1007/s10926-012-9389-6

28. Corbière M, Negrini A, Durand M-J, et al (2017) Development of the Return-to-Work Obstacles and Self-Efficacy Scale (ROSES) and Validation with Workers Suffering from a Common Mental Disorder or Musculoskeletal Disorder. J Occup Rehabil 27:329–341. https://doi.org/10.1007/s10926-016-9661-2

29. Haldorsen EMH, Indahl A, Ursin H (1998) Patients with low back pain not returning to work - A 12-month follow-up study. Spine (Phila Pa 1976) 23:1202–1207. https://doi.org/10.1097/00007632-199806010-00004

30. Hagen EM, Svensen E, Eriksen HR (2005) Predictors and modifiers of treatment effect influencing sick leave in subacute low back pain patients. Spine (Phila Pa 1976) 30:2717–2723. https://doi.org/10.1097/01.brs.0000190394.05359.c7

31. Ekberg K, Wahlin C, Persson J, et al (2015) Early and Late Return to Work After Sick Leave: Predictors in a Cohort of Sick-Listed Individuals with Common Mental Disorders. J Occup Rehabil 25:627–637. https://doi.org/10.1007/s10926-015-9570-9

32. Jensen AGC (2013) A two-year follow-up on a program theory of return to work intervention. Work 44:165–175. https://doi.org/10.3233/WOR-121497

33. Netterstrøm B, Eller NH, Borritz M (2015) Prognostic Factors of Returning to Work after Sick Leave due to Work-Related Common Mental Disorders: A One- and Three-Year Follow-Up Study. Biomed Res Int 2015:1–7. https://doi.org/10.1155/2015/596572

34. Kuijer PPFM, Gouttebarge V, Wind H, et al (2012) Prognostic value of self-reported work ability and performance-based lifting tests for sustainable return to work among construction workers. Scand J Work Environ Heal 38:600–603. https://doi.org/10.5271/sjweh.3302

35. van der Giezen AM, Bouter LM, Nijhuis FJN (2000) Prediction of return-to-work of low back pain patients sicklisted for 3-4 months. Pain 87:285–294. https://doi.org/10.1016/S0304-3959(00)00292-X

36. Dionne CE, Bourbonnais R, Fremont P, et al (2005) A clinical return-to-work rule for patients with back pain. Can Med Assoc J 172:1559–1567. https://doi.org/10.1503/cmaj.1041159

37. Schultz IZ, Crook JM, Berkowitz J, et al (2002) Biopsychosocial Multivariate Predictive Model of Occupational Low Back Disability. In: Spine. pp 191–202

38. Schultz IZ, Crook J, Meloche G., et al (2004) Psychosocial factors predictive of occupational low back disability: towards development of a return-to-work model. Pain 107:77–85. https://doi.org/10.1016/j.pain.2003.09.019
